# Characterization of Inactivated Influenza Vaccines Used in the Russian National Immunization Program

**DOI:** 10.3390/vaccines8030488

**Published:** 2020-08-30

**Authors:** Mikhail Tarasov, Andrei Shanko, Larisa Kordyukova, Anton Katlinski

**Affiliations:** 1 Research and Development Department, FORT LLC, 119435 Moscow, Russia; tarasov-aspirant@yandex.ru; 2 Ivanovsky Institute of Virology, N. F. Gamaleya Federal Research Center for Epidemiology and Microbiology, 123098 Moscow, Russia; 3 Belozersky Institute of Physico-Chemical Biology, Lomonosov Moscow State University, 119991 Moscow, Russia; kord@belozersky.msu.ru; 4 Board Member, FORT LLC, 119435 Moscow, Russia; info@fort-bt.ru

**Keywords:** inactivated influenza vaccines, proteomics, mass spectrometry, SDS-PAGE, dynamic light scattering, electron microscopy

## Abstract

Background: today’s standard quality control methods used to control the protein composition of inactivated influenza vaccines only take into account a few key reference components. They do not allow for thorough characterization of protein compositions. As a result, observation of unpredictable variations in major viral constituents and admixtures of cellular proteins within manufactured vaccines that may seriously influence the immunogenicity and safety of such vaccines has become a pressing issue in vaccinology. This study aims at testing a more sophisticated approach for analysis of inactivated split influenza vaccines licensed in the Russian Federation. The formulations under study are the most available on the market and are included in the Russian National Immunization Program. Methods: liquid chromatography with tandem mass spectrometry (LC-MS/MS) analysis, in combination with label-free protein quantitation via the intensity-based absolute-quantitation (iBAQ) algorithm, as well as a number of standard molecular analysis methods, such as sodium dodecyl sulfate polyacrylamide gel electrophoresis (SDS-PAGE), dynamic light scattering (DLS), and negative-stain transmission electron microscopy (TEM) were applied. Results: the methods implemented were able to identify dozens of viral and host proteins and quantify their relative amounts within the final formulations of different commercially available inactivated split influenza vaccines. Investigation of molecular morphology of the vaccine preparations using TEM revealed typical rosettes of major surface proteins (hemagglutinin and neuraminidase). DLS was used to demonstrate a size distribution of the rosettes and to test the stability of vaccine preparations at increased temperatures. Conclusions: a holistic approach based on modern, highly productive analytical procedures was for the first time applied for a series of different commercially available inactivated split influenza vaccines licensed in Russia. The protocols probed may be suggested for the post-marketing quality control of vaccines. Comparison of different preparations revealed that the Ultrix^®^ and Ultrix^®^ Quadri vaccines produced by pharmaceutical plant FORT LLC and trivalent vaccine Vaxigrip^®^ produced by pharmaceutical company Sanofi Pasteur have well-organized antigen rosettes, they contain fewer admixture quantities of host cell proteins, and demonstrate good correlation among mostly abundant viral proteins detected by different methods.

## 1. Introduction

There are an estimated one billion cases of seasonal influenza reported globally every year. These annual epidemics are estimated to result in about three to five million cases of severe respiratory symptoms, leading to 290,000 to 600,000 deaths [1,2]. Vaccination remains the most effective way to control seasonal influenza [3]. Therefore, the World Health Organization (WHO) recommends annual vaccination against seasonal influenza for healthcare workers and high-risk groups: pregnant women, children aged 6–59 months, the elderly, and persons with specific chronic medical conditions [4]. Many countries run their own national seasonal influenza immunization programs strictly under WHO guidelines [5]. However, scientific periodicals still offer insufficient analytical information on the composition of already licensed products on the market. New sophisticated protocols introduced into practice just recently are based on label-free quantitative mass spectrometry. Yet, they have not been used to study commercially available inactivated vaccines. Currently, the hemagglutinin (HA)-antibody level in a human body is evaluated after immunization to determine the efficacy of vaccines. As far as quality control is concerned, it uses several tests, some of which are semi-quantitative in nature. In this regard, impurities such as substrate cell proteins, which are the major causes of side effects at immunization, remain undetectable or are masked by addition of polymers or adjuvants. Their abundance remains uninvestigated. In the Russian Federation, vaccination against influenza is included in the National Immunization Program (NIP) Schedule; NIP vaccines are only supplied by national manufacturers. A relatively small number of vaccines are procured from global manufacturers abroad, i.e., outside the NIP framework. According to the Global Action Plan for Influenza Vaccines (GAP) [6], the global seasonal influenza vaccine production was 1.467 billion doses in 2015, of which 88% were inactivated influenza vaccines (IIVs) produced through influenza virus replication in the allantoic cavity of chicken embryos (i.e., egg-based vaccines) [7].

Seasonal influenza vaccines include influenza A viruses, subtypes H1N1 and H3N2, and influenza B viruses, Yamagata and Victoria lineages. Trivalent vaccines include one of the influenza B lineages, and tetravalent vaccines include both influenza B lineages [8,9]. However, due to antigenic drift in surface glycoproteins hemagglutinin (HA) and neuraminidase (NA) of the influenza virus, the strains of these types of viruses circulating in the human populations are constantly changing. The WHO annually updates the list of strains recommended for inclusion in seasonal influenza vaccines based on the monitoring of strains circulating in the population in order to maintain the relevance of the antigenic composition of seasonal vaccines [10]. 

In accordance with recommendations, licensed laboratories produce candidate vaccine viruses (CVVs), high-growth reassortant viruses adapted for replication in a production substrate and carrying the surface antigens (HA and NA) of the recommended strains. The success of CVV adaptation to replication in chicken embryos may vary significantly depending on the virus strain and production year. Therefore, vaccine manufacturers may face the challenge of insufficient efficiency of vaccine antigen production [11]. In addition to the growth rate in chicken embryos, CVVs may vary in terms of sensitivity to inactivating and cleaving agents, as well as conditions of virion concentrations and treatment of the virus-containing fluid. All the general steps in the downstream processing of split-virion IIVs (shown in Table S1) determine the content of HA, other viral proteins, and cellular substrate proteins (i.e., chicken (*Gallus gallus*) proteins) within the vaccine, as well as the microstructure of molecular aggregates that are part of the vaccine [12]. These characteristics, therefore, determine vaccine immunogenicity and safety. Possible variability of the protein composition of commercial IIVs highlights the need for control upon entry into the market (i.e., post-marketing control). 

The main idea of the protective immunity elicited by the vaccine comes from production of HA-specific antibodies that inhibit the receptor-binding activity of HA (hemagglutination-inhibiting antibodies) [13]. Thus, the current WHO guidelines for controlling the final bulk-protein composition (i.e., the step immediately preceding the filling process) of IIVs include three tests: determining the HA, total protein, and ovalbumin contents of each strain of the influenza virus [14]. Depending on the manufacturing year and the manufacturer, relative amounts of NA, matrix protein (M1), and nuclear protein (NP) (as well as the immune responses induced by them) in different commercially available inactivated split vaccines may vary significantly, from several up to dozens of times [15]. Thus, new sophisticated platforms for quick and efficient characterization of the manufactured vaccine products should be launched.

Recently, various mass spectrometric approaches for qualitative and quantitative analyses of the proteomes of influenza vaccines have been shown to be feasible. Creskey et al. [16] showed that liquid chromatography and tandem mass spectrometry (LC-MS/MS) may be used to analyze the amino acid sequences of HA and NA in trivalent inactivated influenza vaccines. Hawksworth et al. [17] revealed that a quantitative proteomic approach based on label-free quantitation of the relative protein content using the LC-MS/MS assay provided a detailed description of the protein composition of live influenza vaccines. Various mass spectrometric approaches for measuring the absolute concentrations of HA and NA in vaccines have also been developed [18,19,20,21,22]. An approach was proposed for measuring the absolute HA concentration in vaccines based on sodium dodecyl sulfate polyacrylamide gel electrophoresis (SDS-PAGE) and a sample-deglycosylation procedure [23] as an alternative to single radial immunodiffusion (SRID) used for measuring HA concentrations in vaccines during the production process [14]. Applying the dynamic light scattering (DLS) technique in combination with transmission electron microscopy (TEM) may also provide valuable data for characterization of influenza vaccine microstructures, that is, measuring the hydrodynamic radii of particles and determining the degree of dispersion of vaccine suspensions [12].

This study is the first that is conducting comparative morphological and proteomic analyses of IIVs licensed in the Russian Federation, the most available on the market and included in the NIP. We tested the applicability and informational content of the label-free LC-MS/MS quantitation of protein content for IIV proteome analysis. As supplementary assays, we used SDS-PAGE of vaccine products, deglycosylated with peptide n-glycosidase F (PNGase F), as well as DLS in combination with TEM. The holistic approach implemented in our study enables us to obtain a necessarily detailed description of the compositions of the studied IIVs quite quickly and independently from reference reagents which could be useful for additional post-marketing control of commercially available inactivated vaccines for prevention of seasonal influenza.

## 2. Materials and Methods 

### 2.1. IIV Samples

This study covered the inactivated split-virion vaccines (IIVs) for prevention of seasonal influenza licensed in Russia. All studied vaccines were produced by growing influenza viruses in chicken embryos. Both trivalent inactivated influenza vaccines (IIV3) and quadrivalent inactivated influenza vaccines (IIV4) were investigated. The list of vaccines studied is given below:

- U3—Ultrix^®^ (IIV3, FORT LLC, Russia);
- U4—Ultrix^®^ Quadri (IIV4, FORT LLC, Russia);
- SGU—SOVIGRIPP^®^ (IIV3, NPO Microgen JSC, Russia);
- SGF—SOVIGRIPP^®^ (IIV3, FORT LLC, Russia);
- GP—Grippol^®^ plus, (IIV3, NPO Petrovax Pharm LLC, Russia); and
- VG—VAXIGRIP^®^ (IIV3, Sanofi Pasteur С.А., France).

The strain composition of all vaccines was consistent with the WHO recommendations for 2019–2020 seasonal influenza vaccines in the Northern Hemisphere. For trivalent vaccines:

- Influenza А virus (H1N1): A/Brisbane/02/2018 (H1N1)pdm09-like virus;
- Influenza А virus (H3N2): A/Kansas/14/2017 (H3N2)-like virus; and
- Influenza B virus: B/Colorado/06/2017-like virus (B/Victoria/2/87 lineage).

For quadrivalent vaccines—additional strain:

- Influenza B virus: B/Phuket/3073/2013-like virus (B/Yamagata/16/88 lineage).

Normative HA content of each subtype of the influenza virus per dose (0.5 mL):

- U3: A (H1N1)—15 μg; А (Н3N2)—15 μg; В (Victoria)—15 μg;
- U4: A (H1N1)—15 μg; А (Н3N2)—15 μg; В (Victoria)—15 μg; В (Yamagata)—15 μg;
- SGU: A (H1N1)—5 μg; А (Н3N2)—5 μg; В (Victoria)—11 μg;
- SGF: A (H1N1)—5 μg; А (Н3N2)—5 μg; В (Victoria)—11 μg;
- GP: A (H1N1)—5 μg; А (Н3N2)—5 μg; В (Victoria)—5 μg;
- VG: A (H1N1)—15 μg; А (Н3N2)—15 μg; В (Victoria)—15 μg.

Vaccines U3, U4, and VG are free of adjuvants and preservatives. Vaccines SGU and SGF contain the SOVIDON^ТМ^ synthetic polymer (a copolymer of 2-methyl-5-vinylpyridine and N-vinylpyrrolidone)—500 μg per dose (0.5 mL). Vaccine GP contains the Polyoxidonium^®^ synthetic polymer (high-polymeric units of 100 kDa based on both N-oxide 1.4-ethylene piperazine and (N-carboxyethyl-) 1.4 ethylene piperazine bromide)—500 μg per dose (0.5 mL). As claimed by the vaccine manufacturers, these polymers act as adjuvants. The SGU vaccine also contains thimerosal (merthiolate) as a preservative—50 μg per dose (0.5 mL). 

### 2.2. Lowry Protein Assay

The total protein content in the vaccines was measured using the Lowry protein assay (Peterson’s modification) with protein precipitation [24] using the Total Protein Kit, Micro Lowry reagent kit (Sigma-Aldrich, Germany, product number: TP0300) in accordance with the manufacturer’s protocol. Optical density was measured at 650 nm on Infinite^®^ 200 PRO plate reader (TECAN, Switzerland) in 96-well plates (Greiner, Kremsmünster, Austria). Optical density was measured in a 200 μL volume of the test solution per well. The optical density of the solution in each well was determined as the average of 25 measurements. Optical densities of all solutions were measured in three independent repeats, and their arithmetic mean was used to draw the calibration curve.

### 2.3. Peptide N-Glycosidase F (PNGase F) Treatment

Vaccine samples as they are (without additional concentration) were processed with PNGase F before they were loaded into gels for SDS-PAGE analysis using a deglycosylation kit manufactured by New England Biolabs (NEB), Ipswich, MA, USA (catalog number P0704L) in accordance with the manufacturer’s protocol. In brief, 1× Glycoprotein Denaturing Buffer (from 10× Stock) (NEB) was added to the vaccine samples, heated to 100 °C, incubated at this temperature for 10 min, cooled in ice for about 10 s, and centrifuged at 15,000× *g* for 10 s. Then, 1× GlycoBuffer 2 (10×) (NEB), 1× NP-40 (10%) (NEB), and PNGase F (NEB) were added to the reaction mix, the latter at a ratio of 5 activity units per μg of total protein in the sample. The reaction mix was subsequently incubated at 37 °C overnight. The processed samples were loaded onto the gels in equal volumes (see SDS-PAGE procedure in Section 2.4).

To increase the coverage of surface N-glycosylated influenza virus glycoproteins HA and NA by MS-identified tryptic peptides, we also treated samples with PNGase F before LC-MS/MS analysis according to [16] with some modifications. For this, aliquots of the vaccine samples containing 10 μg of total protein were selected (based on preliminary quantitation of total protein by the Lowry protein assay, as described in Section 2.2). The volumes were brought to 100 μL with water (MS grade). Because of the low protein content in the SGU and SGF samples, 10-fold concentrates obtained via centrifugation through 5 kDa molecular weight cut-off filters Vivaspin Turbo 4 (Sartorius, Göttingen, Germany) were used. ProteaseMax (Promega, Madison, WI, USA) 0.05%, acetonitrile 5% (Sigma Aldrich, St. Louis, MO, USA), and triethylammonium bicarbonate 50 mM (Sigma Aldrich, USA) were added to each sample (final concentrations). The reaction mix volume was brought to 200 μL with water, heated to 100 °C and maintained at this temperature for 10 min, then cooled in ice for 10 s, after which 0.1 μL (50 activity units) of PNGase F (NEB) was added to each sample. The reaction mix was then incubated at 37 °C overnight. The reaction mix was then transferred to the sample-preparation stage for LC-MS/MS analysis.

### 2.4. SDS-PAGE

Denaturing one-dimensional electrophoresis was performed using sodium dodecyl sulfate (SDS)—polyacrylamide gel electrophoresis with a 12.5% concentration of acrylamide (Sigma Aldrich, USA) in the separating gel and 5% concentration in the stacking gel [25,26]. Combs with a 5 mm tooth width were used to form pockets for the samples. In order to reduce disulfide bonds, the analyzed samples were placed in the sample buffer (2% lithium dodecyl sulfate (LDS), 0.065 M Tris-HCl (pH 6.8), 1% dithiothreitol (DTT), 10% glycerol, and 0.01% bromophenol blue (all from Sigma Aldrich, USA)) and were heated in a boiling water bath for 2 min. An amount of 1 μL of the Precision Plus Protein Standard (BioRad, Hercules, CA, USA) was loaded as a marker. A maximum of 40 μL (32 μL of the analyzed sample and 8 μL of the buffer) was applied to the lane. Electrophoretic separation (SDS-PAGE) of the sample proteins was performed using the Hoefer miniVE system (Hoefer Inc., Holliston, MA, USA) (gel size of 80 × 90 × 1 mm). Gels were stained with Coomassie Blue R-350 (GE Healthcare, USA) for 2 h.

### 2.5. LC-MS Quality Control Mix

In order to verify label-free LC-MS/MS-based quantitative analysis to correctly determine the protein ratio in a complex multicomponent mix, we used the LC-MS quality control (QC) mix that was added to each of the studied vaccine samples before the start of mass spectrometric analysis (MSA). It was already shown that QC mix is effective in verifying the label-free LC-MS/MS assessment of protein ratios at different stages of production of live attenuated influenza vaccines [17]. The LC-MS QC mix that we used included four human proteins (control proteins) and four synthetic stable-isotope-labeled (SIL) peptides, each corresponding to one tryptic peptide from one control protein, isolated from blood plasma: fibrinogen (equimolar mix of three subunits FIBA, FIBB, FIBG (in our study, we focused on two, FIBB and FIBG) bound in the native protein by disulfide bonds that break down under reducing conditions), apolipoprotein A1 (APOA), and apolipoprotein B (APOB) (Table S2). Lyophilized preparations of these proteins were purchased from IMTEK (Russia). Next, we a used high-resolution (HR) liquid chromatography-mass spectrometry (LC-MS) assay to measure the absolute concentration of each control protein in accordance with the approach described by Guo et al. [22] (see Section 2.8). The obtained ratios of absolute concentrations of the control proteins were compared with ratios of their abundance obtained via label-free LC-MS/MS-based calculations using the intensity-based absolute-quantitation (iBAQ) algorithm [27].

The lyophilized proteins were dissolved in MS-grade water to a 2 mg/mL concentration. The resulting stock solutions were stored at –20 °C. Then, protein solutions were quantitatively added to each sample of the studied vaccines after PNGase F treatment and before the reduction/alkylation step (the volume of each sample contained 10 μg of the total vaccine protein and did not exceed 100 μL). Concentrations of control proteins in the vaccine samples were selected such that the minimal concentration of the control protein (APOB) was about 10 times lower than the total HA normative concentration, and the maximum (APOA) was several times higher than the total HA normative concentration (Table 1). Thus, the concentration range covered by the control proteins was ~1.5 to 2 orders of magnitude. It included the concentrations of the target vaccine proteins.

Selection, synthesis, and determination of the concentrations of SIL peptides were carried out by the Human Proteome Center, Institute of Biomedical Chemistry (IBMC), Moscow, Russia, in accordance with the method described by Kopylov et al. [28]. Concentrations of synthetic peptides were measured using fluorescent-signal detection of the amino acid derived after peptide acidic hydrolysis. SIL peptides were added in known concentrations to the test vaccine samples one hour before trypsin digestion (see trypsinolysis conditions in Section 2.6). Concentrations of SIL peptides in the vaccine samples were selected such that they did not differ from standard protein concentrations by more than two orders of magnitude (Table 1).

### 2.6. Reduction, Alkylation, and Trypsin Digestion

The sample was prepared for MSA in accordance with the filter-aided sample preparation (FASP) protocol [29]. In order to reduce and alkylate disulfide bonds, samples were incubated in 4 mM tris (2-carboxyethyl) phosphine (TCEP; Sigma Aldrich, USA) and 6.2 mM 2-chloroacetamide (CAA; Sigma Aldrich, USA) at 80 °С for 30 min. Then, the reaction mix was placed in Microcon YM-10 centrifugal filter units (Merck Millipore, Burlington, MA, USA) and centrifuged at 11,000× *g* for 15 min in a thermostatic centrifuge at 20 °C. The samples were rinsed three times by adding 200 μL of the buffer containing 50 mM of tetraethylammonium bicarbonate (pH = 8.5), followed by centrifugation at 11,000× *g* for 15 min in a thermostatic centrifuge at 20 °C.

To hydrolyze proteins of the vaccine preparations after the last rinsing, 50 μL of the buffer containing 50 mM tetraethylammonium bicarbonate (pH = 8.5) and trypsin (Promega, USA) with an enzyme/total protein weight ratio of 1/50 were added to the samples. The mix was incubated overnight at 37 °C, with 350 rpm shaking. Samples were centrifuged on filters at 11,000× *g* for 15 min in a thermostatic centrifuge at 20 °C to obtain a peptide solution. Then, the filters were rinsed with 50 μL of a 30% solution of formic acid (Sigma Aldrich, USA) by centrifugation at 11,000× *g* for 15 min in a thermostatic centrifuge at 20 °C. The filtrate was dried in a vacuum concentrator and dissolved in 20 μL of 5% formic acid for subsequent MSA.

### 2.7. LC-MS/MS

LC-MS/MS was performed using the Ultimate 3000 RSLCnano chromatographic HPLC system (Thermo Scientific, Waltham, MA, USA) coupled with a Q Exactive HF-X mass spectrometer (Thermo Scientific, USA). We loaded 1 μg of the peptide mix onto the Acclaim μ-Precolumn enrichment column (0.5 × 3 mm, 5 μm particle size; Thermo Scientific, USA) at a flow rate of 10 μL per minute for four minutes in isographic mode using buffer C as a mobile phase (2% acetonitrile (Sigma Aldrich, USA), 0.1% formic acid in deionized water). Peptides were then separated on the Acclaim Pepmap^®^ C18 HPLC column (75 μm × 150 mm, 2 μm particle size; Thermo Scientific, USA) in gradient-elution mode. The gradient was formed by mobile phases A (0.1% formic acid) and B (80% acetonitrile, 0.1% aqueous solution of formic acid) at a flow rate of 0.3 μL per minute. The column was rinsed with 2% mobile phase B for four minutes, with subsequent a linear increase in mobile phase B concentration to 35% in 45 min and a linear increase in phase B concentration to 99% in 5 min; after 10 min rinsing with 99% buffer B, the concentration of this buffer was linearly reduced to the initial level of 2% in six minutes. Overall, the analysis took 70 min.

MSA was performed on a Q Exactive HF-X mass spectrometer (Thermo Scientific, USA) in positive-ionization mode using the NESI (nanoelectrospray) source. The MSA was performed under 2.1 kV emitter voltage and 240 °C capillary temperature. Panoramic scanning was carried out in a mass range of 300 to 1500 *m*/*z* with a 120,000 resolution. In tandem scanning, resolution was set to 15,000 in a 100 *m*/*z* mass range to the upper limit, which was automatically determined based on the precursor mass, but of no more than 2000 *m*/*z*. Precursor-ion isolation was performed in a window of ±1 Da. The maximum number of ions allowed for isolation in the MS2 mode was set to be within 40. In addition, 50,000 units was set as the cut-off limit for selecting the precursor for tandem analysis, while the normalized collision energy (NCE) was equal to 29. Only ions with charges from z = 2+ to 6+ were considered for tandem scanning. Maximum accumulation time was 50 ms for precursor ions and 110 ms for fragment ions. The AGC (automatic gain control) value for precursors and fragment ions was set to 1 × 106 and 2 × 105, respectively. All measured precursors were dynamically excluded from tandem MS/MS analysis for 90 s. Each sample was analyzed in three runs, and the results were used for further bioinformatics processing.

### 2.8. Data Processing

The densitometry of protein bands was determined using ImageMaster™ 2D Platinum 7.0 software (GE Healthcare, Chicago, IL, USA). Molecular-weight values for protein bands were determined using Precision Plus Protein Standards (BioRad, Hercules, CA, USA). The relative volume of the protein spot (% vol) value was used to assess protein weight in the band on the gel. To evaluate the relationship between total HA weight and total protein weight in the vaccine (relative HA weight, %), we summed the % vol of the HA1 and HA2 bands (after sample N-deglycosylation) on the lane and divided the value by the sum of the % vol of all bands of the same lane. The absolute weight of total HA was calculated as the relative HA weight of the total protein weight measured by the Lowry protein assay (with protein precipitation).

Mass spectra were analyzed using MaxQuant 1.5.8.3 software [30] in accordance with the Hutchinson and Stegmann protocol [31]. Files with the mass-spectrometric analysis results for all three runs for each sample were analyzed together using standard settings and the following parameters. Enzyme, trypsin/P; variable modifications, oxidation (M) and acetyl (Protein N-ter); fixed modifications, carbamidomethyl (C).

Peptide spectra were matched to a database containing the reference chicken (*Gallus gallus*) proteome (UniProt database UP000000539 (https://www.uniprot.org/proteomes/UP000000539)) and the expected proteomes of virions of the influenza virus strains recommended by the WHO for inclusion in 2019–2020 seasonal influenza vaccines in the Northern Hemisphere. The lists of expected virion proteins were compiled based on recent studies on mass spectrometric analysis of influenza virions [32] and live attenuated influenza vaccines [17]. Viral protein sequences for inclusion in the database were consensus sequences generated by a computer algorithm (Geneious Prime 2019.8.1.9 (Biomatters, Auckland, New Zealand)) on the basis of multiple alignments of amino acid sequences of viral proteins of the isolates of influenza virus strains with completely sequenced genomes uploaded to the EpiFlu^®^ GISAID public database [17,33]. We also included PNGase F, trypsin, and human protein sequences in our database from the LC-MS QC mix. All proteins belonging to the automatically generated base of decoy proteins (reverse sequences) were excluded from the identified proteins.

The iBAQ value was used for label-free quantitation of protein abundance [27]. For the quantitation of vaccine proteomes, all mass spectrometrically identified proteins were grouped in accordance with genes encoding these proteins, namely, HA A, HA B, NA, NP, M1, M2, non-structural protein one (NS1), nuclear export protein (NEP), three viral polymerase subunits - PB1, PB2, PA, and NB. Justification of the viral-protein grouping principle is given in Section 3.3.

Absolute concentrations of total HA in the vaccines were calculated using label-free LC-MS/MS analysis and based on the fact that the iBAQ value of the protein was directly proportional to the amount of substance of this protein. The proportionality factor was the same for all proteins from one sample [27]. The algorithm for calculating the HA concentration was similar to that based on the densitometry results. First, we calculated the relative weight of total HA (relative HA weight, %) as the ratio of the sum of products of the HA iBAQ of each strain multiplied by their molecular weights to the sum of products of the iBAQ of all proteins in the sample multiplied by their molecular weights:

(1) $$\mathrm{relative}\;\mathrm{HA}\;\mathrm{weight},\%=\frac{\sum^{\;}\left[\mathrm{iBAQ}\left({\mathrm{HA}}_{i}\right)\;\times \;\mathrm{MW}({\mathrm{HA}}_{i})\right]}{\sum^{\;}\left[\mathrm{iBAQ}\left({\mathrm{prot}}_{k}\right)\;\times \;\mathrm{MW}\;\left({\mathrm{prot}}_{k}\right)\right]}\times 100,$$

where Σ is the summation symbol; $\mathrm{iBAQ}\left({\mathrm{HA}}_{i}\right)$, HA iBAQ is the value of one influenza virus strain of the studied sample; I the is influenza virus-strain number; $\mathrm{MW}({\mathrm{HA}}_{i})$ is the molecular weight of influenza virus strain HA (molecular weights of deglycosylated HA were used); $\mathrm{iBAQ}\left({\mathrm{prot}}_{k}\right)$, iBAQ is the value of the protein of the studied sample; and k is the protein number of the studied sample. Absolute concentration of total HA was calculated using the following formula:

(2) $$\mathrm{HA}\;\mathrm{concentration}=\frac{\mathrm{relative}\;\mathrm{HA}\;\mathrm{weight},\%\;\times \;\mathrm{total}\;\mathrm{protein}}{100},$$

where total protein (μg/mL) was measured via the Lowry protein assay.

Absolute concentrations of control proteins were determined by the corresponding SIL peptides by the (HR) LC-MS assay in accordance with the protocol validated by Guo et al. [22]. Extracted ion chromatograms of natural tryptic peptides and corresponding SIL peptides were generated. Chromatograms were analyzed using XCalibur 3.1 software (Thermo Scientific, USA) supplied with the mass spectrometer. The following settings were used for automatic peak detection and integration: peak-integration algorithm, ICIS; smooth points, 11; baseline window, 100; minimal peak height (S/N), 3; mass tolerance, 5 ppm. Ratios of areas under the peaks of the corresponding natural and synthetic peptides were interpreted as the ratios of their concentrations. As was shown in the study by Guo et al. [22], one representative peptide is sufficient for accurate measurement of protein concentration. Thus, concentrations of tryptic peptides obtained were equated with concentrations of the starting proteins from which these tryptic peptides were derived.

### 2.9. Dynamic Light Scattering (DLS)

DLS particle-size distributions in the samples were measured by means of a Zetasizer Nano ZS (Malvern Panalytical, Malvern, UK) at a fixed scattering angle of 173° in a thermostatic cell at 25 °C. We used 100 averages of 25 s scans to obtain a histogram with ~5% accuracy. Data were analyzed using Dispersion Technology software, version 5.10 (Dispersion Technology, Inc., Bedford Hills, NY, USA).

### 2.10. Negative-Stain Transmission Electron Microscopy

Vaccine solutions were deposited on formvar–carbon-coated grids (TED Pella, Redding, CA, USA), incubated for two minutes. After that, excess solution was removed, and the samples were then stained for 20 s with a 2% water solution of phosphotungstic acid (PTA; Sigma Aldrich, USA), pH 7.0. Samples were viewed with a transmission electron microscope Jeol JEM-2100 (JEOL Ltd., Tokyo, Japan).

## 3. Results

### 3.1. IIV Protein Separation by SDS-PAGE

**Figure 1:**
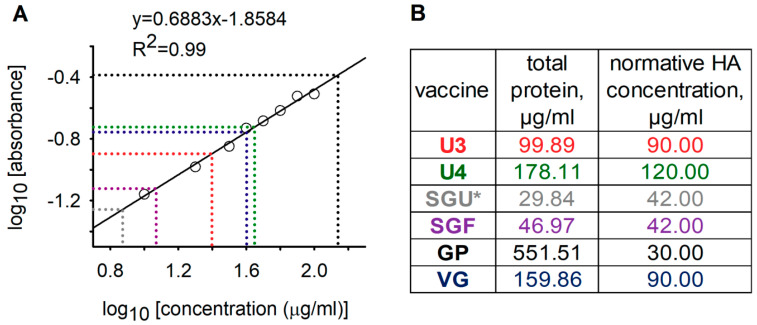
Total protein quantitation in vaccine samples based on the Lowry protein assay (Peterson’s modification) with protein precipitation. (**A**) Calibration plot describing the dependence of the decimal logarithm of light absorption (at 650 nm) on the decimal logarithm of protein concentration. Circles represent the values of standard bovine-serum-albumin (BSA) solutions; solid line and equation above it represent the linear approximation of experimental values of standard BSA solutions; R^2^ is the ratio of determination of the linear approximation of experimental values; dashed lines are the dependence of total protein concentrations in vaccines on their optical density. Total protein measured in fourfold dilutions of the original vaccines such that their concentrations are close to the concentration range of the calibration BSA solutions. (**B**) Table showing the results of the total protein measurements in original vaccines and normative concentrations of hemagglutinins (HAs) in accordance with instructions for vaccines. Vaccine colors correspond to dashed-line colors on the calibration chart. SGU is marked with an asterisk since the measured total protein in the vaccine was less than the normative HA concentration.

In accordance with WHO recommendations for inactivated-influenza vaccine (IIV) production and control, the total protein in vaccines was quantified using the Lowry protein assay with protein precipitation [14]. Our quantitation of total protein via the Lowry protein assay (Peterson’s modification) with protein precipitation [24] showed that the total protein concentration in vaccines with synthetic polymers (SGF, SGU, GP) was critically different from in vaccines without synthetic polymers (U3, U4, VG) (Figure 1). Since a wide range of substances may interfere with protein quantitation by the Lowry protein assay [34], we assumed that the abnormal total protein in the SGF, SGU, and GP vaccines may be due to interference of the SOVIDON^ТМ^ and Polyoxidonium^®^ polymers with the Lowry protein assay.

**Figure 2:**
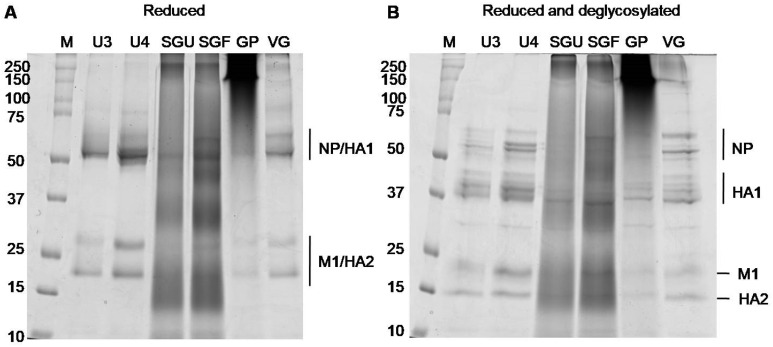
SDS-PAGE of IIV samples. Vaccine samples applied (**A**) after reduction and (**B**) after reduction and peptide n-glycosidase F (PNGase F) treatment; M, marker. An amount of 32 μL of initial suspension of each preparation was applied to each lane. Deglycosylation-improved protein cleavage. Glycosylated hemagglutinin (HA)1 (~64 to 79 kDa) was localized close to NP (~55 to 66 kDa). Glycosylated HA2 formed a band at the ~23 to 25 kDa level close to band M1 (~26 kDa). After deglycosylation, the HA1 band shifted to the ~37 to 47 kDa level, and HA2 to ~23 kDa (experimental molecular weights are consistent with previous studies [11,15,26]).

We used the SDS-PAGE assay to obtain more detailed information on the protein composition of the analyzed IIVs. A number of published papers have shown that a fruitful approach utilizes denaturing gel electrophoresis preceded by the procedure of disulfide bond reduction (by heating with reducing agents, for example, dithiothreitol (DTT), which leads to separation of HA0 into HA1 and HA2) and gives a characteristic reproducible pattern of protein-band distribution in gels [12,23,35]. Using the reducing SDS-PAGE protocol, we conducted a comparative analysis of all six commercially available vaccines to characterize their protein composition. Equal volumes of initial suspensions (32 μL) were applied to each lane. As can be seen from Figure 2, SDS-PAGE analysis revealed diffuse dying of the whole lane, and the major protein bands were not clearly detected in the case of SGF, SGU, and GP vaccine samples. This is probably due to the presence of synthetic polymers with high molecular weight (500 μg per dose (0.5 mL)) within these vaccine samples possibly causing protein aggregation and not allowing separation amidst SDS (Figure 2 and Figure S1). For vaccines without synthetic polymers (U3, U4, VG), we were able to reproduce the expected patterns of viral proteins before and after the preliminary deglycosylation (Figure 2 and Figure S1).

Protein bands that appeared after deglycosylation within the range of 37 to 44 kDa were used to measure the HA1 concentration, and the band that appeared below band M1 after deglycosylation was used to measure the HA2 concentration. The densitometry results obtained for the gels shown in Figure S1A,B are represented in Tables S2 and S3. The correlation between HA concentrations measured by the SDS-PAGE assay for the U3, U4, and VG vaccine samples and normative HA concentrations (recovery, %) varied from 69.23% to 75.71% (Table 2), which approximately corresponded to the previously described degree of proximity of SRID and SDS-PAGE results [11,15,26].

### 3.2. Verification of Label free LC-MS/MS-Based Protein Quantitation in IIV Samples

For the purpose of reliable quantitative analysis of IIV protein composition using label-free LC-MS/MS, we checked whether the iBAQ value calculated in MaxQuant software using the results of label-free LC-MS/MS analysis correctly determined the ratio of proteins in IIVs.

Therefore, we mixed vaccine samples with protein standards (LC-MS QC mix). Subsequently, LC-MS/MS analysis of vaccines with added protein standards was carried out on an Orbitrap-based mass spectrometer (Q Exactive HF-X (Thermo Scientific, USA)), which allowed for quantitative assessment of the content of the control proteins using two orthogonal methods: (1) (HR) LC-MS assay: measurement of absolute protein concentrations by integrating the peaks of natural and corresponding SIL peptides in the extracted ion chromatograms obtained by high-resolution (HR) LC-MS analysis of the peptide mix (Figures S2–S19); (2) shotgun-proteomics assay: calculation of iBAQ (a sum of intensities of MS peaks for all identified peptides of a given protein, normalized to the number of theoretically predicted peptides of the protein) based on the results of LC-MS/MS of the peptide mix and subsequent identification of proteins using a theoretically predicted protein sequence database (Figure S20). Within the framework of our study, we considered the (HR) LC-MS assay as a gold standard reflecting the true protein content, since Guo et al. [22] showed this method to have high accuracy and reliability.

Results of the ratio of control proteins based on iBAQ and of their absolute concentrations are summarized in Figure S20. This iBAQ level of accuracy for low-represented proteins is consistent with published results [17]. The dynamic range of concentrations covered by control proteins varied from 1.5 to 3 orders of magnitude. It included the total concentrations of all viral proteins (calculated on the basis of iBAQ). Moreover, the total content of viral proteins was approximately in the middle of the dynamic range of the control protein concentration in all vaccine samples. Therefore, this dynamic range was relevant for verification of iBAQ evaluation of the protein content in vaccines within the range of at least ±1 order of the total content of all viral proteins.

**Figure 3:**
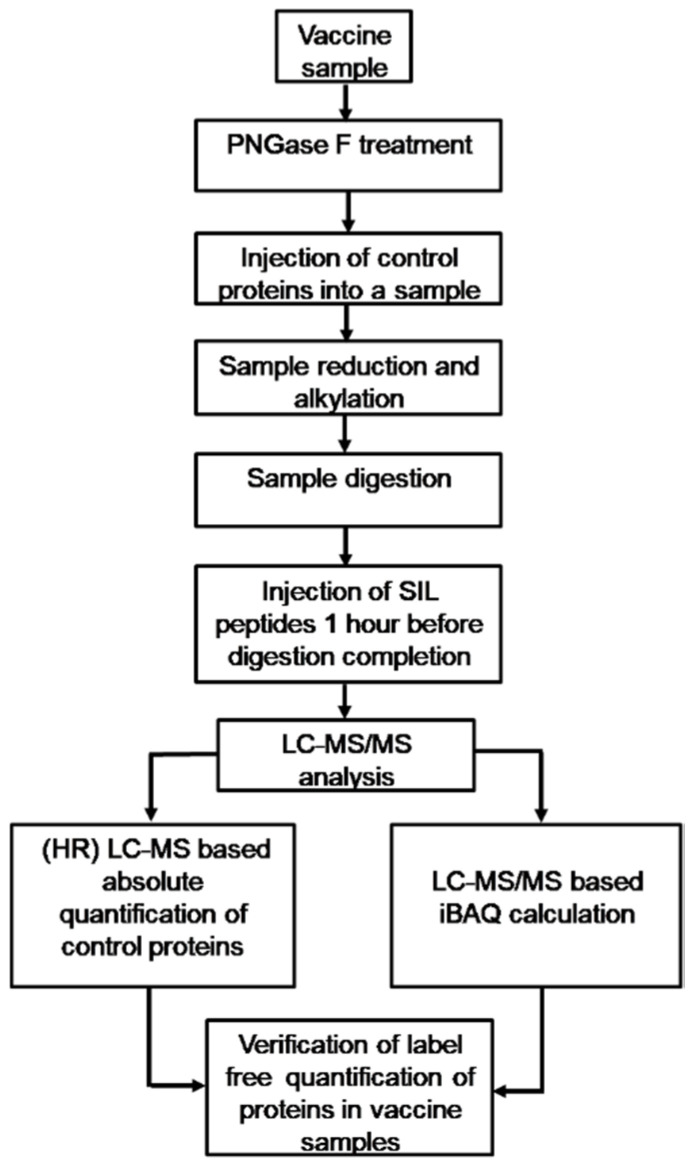
Workflow chart showing the general stages involved in mass spectrometric quantitative proteomics analysis of inactivated influenza vaccines (IIVs).

Since LC-MS/MS does not require the conditions to be optimized for different proteins and since control proteins were mixed with vaccine samples for our study prior to sample preparation for LC-MS/MS analysis (Figure 3), meaning that they were processed simultaneously with the vaccine proteins by the same protocols, we concluded that the iBAQ calculation accuracy for vaccine protein content corresponded to the iBAQ calculation accuracy for the control protein content in the above concentration range (±1 order of the total content of all viral proteins). So, the MSA tools and protocols used in our study enabled us to conduct a fairly reliable quantitative comparison of IIV proteomes.

### 3.3. Label-Free LC-MS/MS-Based Quantitation of Proteins in IIV Samples

Based on peptide mass spectra with fragmentation patterns (LC-MS/MS assay) and subsequent computer search for peptide spectrum matches (PSMs) in the theoretically predicted protein database, we compiled lists of identified proteins and their corresponding iBAQ values in six commercially available IIVs (see MaxQuant reports in Supplementary Documents 2–7).

The results of HA and NA strain identification and the corresponding sequencing coverage in the studied vaccines are summarized in Table 3. The table shows that the LC-MS/MS assay with preliminary deglycosylation of HA and NA enabled us to obtain a sufficient percentage of peptide coverage of these glycoproteins in order to confirm the presence of two influenza type B virus strains in tetravalent vaccine U4 and one type B virus strain in the trivalent vaccines. At the same time, the percentage of the SGU and GP sample coverage was several times lower than that in other vaccines. This was probably due to differences in the process workflow; the coverage percentage also depends on (1) the extent of consistency between theoretically predicted and actual amino acid sequences of proteins, (2) the absolute amount of proteins in the analyzed samples, and (3) the sample preparation conditions for LC-MS/MS.

For quantitative proteomic analysis, we grouped the identified viral proteins such that homologous proteins belonged to the same group, and nonhomologous proteins to different groups. Thus, the list of viral protein groups was HA A, HA B, NA, NP, M1, M2, NS1, NEP, PA, PB1, PB2, NB. This technique was used because of the incomplete coverage of proteins with the peptides identified via mass spectra [16] and the high mutability of the influenza virus [36]. Both factors may lead to incorrect iBAQ calculation for homologous proteins due to computer-algorithm errors in the distribution of identified peptides among proteins.

**Figure 4:**
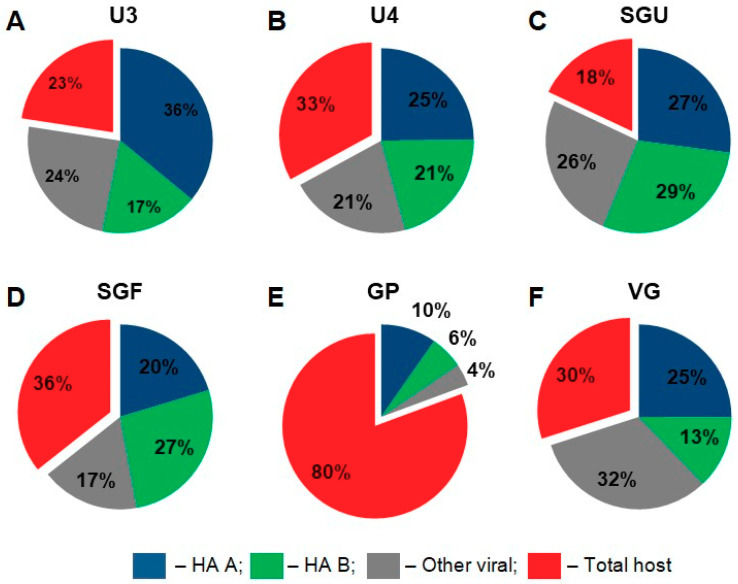
Label free LC-MS/MS-based quantitation of general protein groups in IIVs. Pie charts are based on the intensity-based absolute-quantitation iBAQ algorithm results. The percentages show the ratio of the designated proteins to total protein measured in each sample. (**A**) U3; (**B**) U4; (**C**) SGU; (**D**) SGF; (**E**) GP; (**F**) VG.

Quantitative proteomic analysis in our study was primarily aimed at comparing the relative content of proteins belonging to main functional protein groups in the vaccines. For this purpose, all identified proteins were divided into four groups: influenza type A virus hemagglutinin (HA A), influenza type B virus hemagglutinin (HA B), other viral proteins, and host proteins (chicken (*Gallus gallus*)). The iBAQ values were summed for the proteins within each group (Figure 4).

The resulting pie charts (Figure 4) show that the protein composition of the studied IIVs varied considerably. In the GP vaccine sample, the total abundance of host proteins was 80% of the total abundance of all detected proteins. In the remaining five vaccines, host proteins ranged from 18% (SGU) to 36% (SGF).

The relative content of HA A and B in all vaccines showed a correlation with the normative concentrations of these protein groups. Namely, HA A was approximately double HA B in the following vaccines: U3 (36% HA A vs. 17% HA B), GP (10% HA A vs. 6% HA B), and VG (25% HA A vs. 13% HA B). The normative HA content in these vaccines was as follows (μg per dose): U3 and VG (30 HA A vs. 15 HA B) and GP (10 HA A vs. 5 HA B). The relative abundance of HA A and B was quite similar in the following vaccines: U4 (25% HA A vs. 21% HA B), SGU (27% HA A vs. 29% HA B), and SGF (20 % HA A vs. 27 % HA B). The normative HA content in these vaccines was as follows (μg per dose): U4 (30 HA A vs. 30 HA B), and SGU and SGF (10 HA A vs. 11 HA B).

To verify the correlation between the absolute quantitation of total HA in vaccines with SDS-PAGE (Table 2) and quantitative MSA, we calculated total HA using iBAQ values, theoretical molecular weights of the corresponding proteins, and measured total protein values in the vaccines (Figure 1; see MaxQuant reports in Supplementary Documents 2–7; for the calculation algorithm, please refer to Section 2.8); the results are presented in Table 4. Discrepancies between the results of these two methods (relative difference, %) varied from 4.83% to 7.51%.

SDS-PAGE (in combination with a preliminary deglycosylation step and densitometry analysis) and label-free LC-MS/MS assays are based on different physical mechanisms. In addition, protein weights used to calculate HA concentrations via proteomic analysis may differ from the actual molecular weights of the proteins due to unaccounted post-translational modifications. Taking these factors into account, we conclude that the semi-quantitative evaluation of total HA deduced from the SDS-PAGE and its absolute quantitation based on the label-free LC-MS/MS were close enough to be considered consistent.

The inapplicability of the Lowry assay to the samples containing large proportions of the synthetic polymers did not allow us to reliably measure the total viral protein within the SGU, SGF, and GP vaccines. Accordingly, applying the LC-MS/MS assay to quantify the absolute concentrations of total HA in these vaccines in comparison with the normative HA content quantified by the Lowry assay had no sense. However, absolute quantitation of HA concentrations with the LC-MS/MS assay was valuable if the vaccine did not contain polymers. Therefore, we compared the percentage correspondence between LC-MS/MS-based and normative values of the absolute concentration of total HA in vaccines without synthetic polymers (U3, U4, and VG; Table 5). Recovery values (%) for vaccines without polymers ranged from 64.57% to 72.04%.

**Figure 5:**
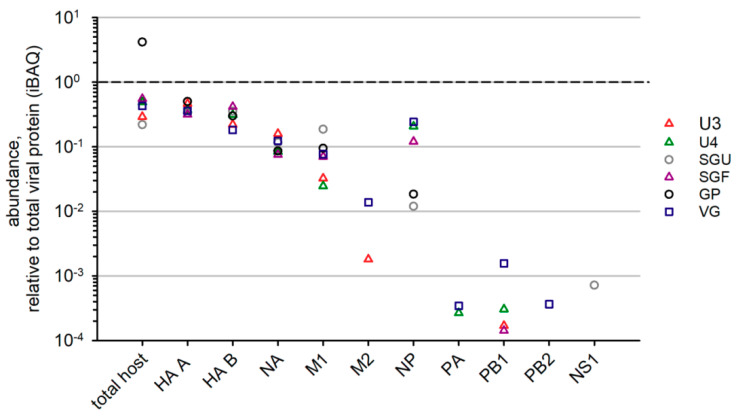
Label-free LC-MS/MS-based quantitation of viral protein groups in IIVs. Protein abundance normalized to the abundance of total viral protein in corresponding preparations.

In our study, for all six IIVs, NA, HA A, and HA B abundance values were quite similar in relation to total viral protein (Figure 5). Among intrinsic virion proteins in IIVs, the most abundant were M1 and NP, which correlated with the relatively high content of these proteins in virions [32]. However, in different vaccines, the content of these proteins varied significantly. We were not able to determine the least abundant virion proteins (M2, NS1, NEP, NB, PB1, PB2, and PA) in all IIVs due to method sensitivity. VG was found to have the most complete list of low-represented virion proteins.

### 3.4. IIV Characterization Using TEM and DLS

We used negative-stain transmission electron microscopy (TEM) and dynamic light scattering (DLS) to evaluate the microstructure of IIVs. In interpreting the TEM images obtained, we used the results of previous electron microscopy studies of the influenza virus [37,38], whole inactivated virus vaccines [39], and purified recombinant HA [40,41], as well as TEM images of influenza virions we derived from the virus-containing allantoic fluid (Figure S21).

**Figure 6:**
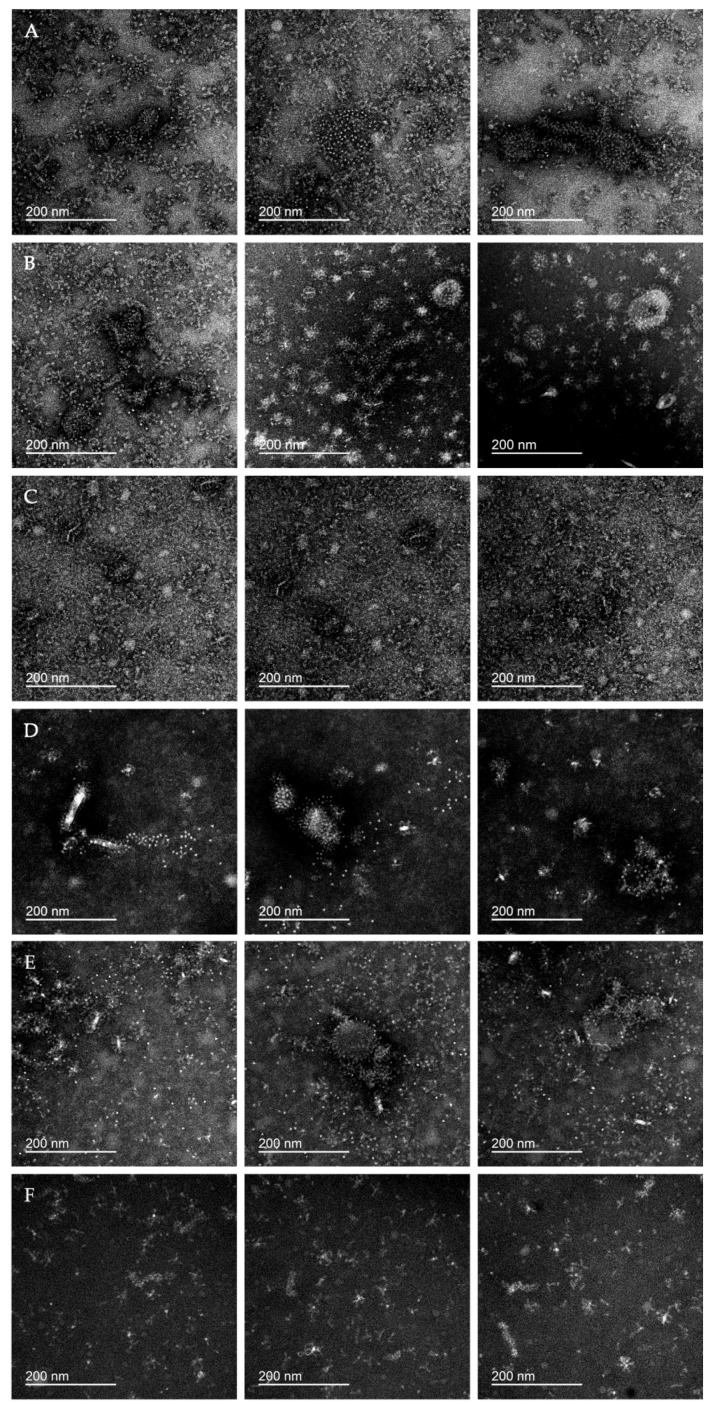
Negative-stain transmission electron microscopy (TEM) of IIV samples. Each row shows three representative images of the vaccine sample; samples (**A**) U3, (**B**) U4, (**C**) SGU, (**D**) SGF, (**E**) GP, and (**F**) VG. Scale bars on all images are 200 nm.

In the TEM images of all vaccine samples (Figure 6), we were able to detect the abundance of structures identical to HA spikes present on the surface of native influenza virions. In the vast majority of cases, visible HA spikes were organized into morphologically diverse HA rosettes that were similar to the molecular aggregates detected on the electron microscopy images of recombinant HA [40,41]. Additional images of representative HA rosettes can be found in Figure S21. A wide variability of molecular aggregates and structures observed may be interpreted as parts of the split virions (e.g., within the U4 and GP samples; Figure 6B,E, center and right panels).

**Figure 7:**
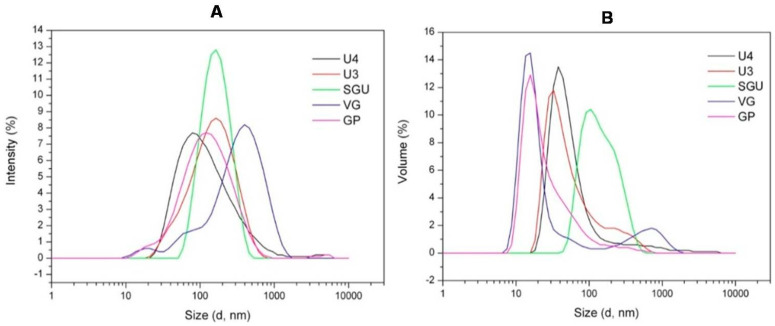
Dynamic light scattering (DLS) assay of IIV samples. Particle-size distribution by (**A**) intensity and (**B**) volume.

This variability of morphological structures detected via the TEM analysis may be caused by different detergent efficiency disrupting the virions as well as different purification protocols applied by different vaccine manufacturers to remove the inner viral proteins (M1 and NP). The Dynamic light scattering (DLS) was used for quantitative analysis of the size of particles in the vaccines. On the basis of DLS measurements of the U3, U4, SGU, and VG samples, particle-size distributions by intensity (Figure 7A) and by volume (Figure 7B) were generated, and the polydispersity index (PDI; Table 6) was calculated. The PDI of the studied vaccines varied in a wide range (from 0.191 (SGU) to 0.54 (VG))—which nevertheless falls into the allowable range of 0.05 (very monodisperse suspension) to 0.7 (very wide particle-size scatter, upper limit of DLS assay sensitivity) [42]. Intensity distribution (Figure 7A) showed that the VG vaccine significantly deviated from other vaccines by a median particle diameter. The median particle diameter in VG was ~300 nm, and no more than 160 nm in the remaining vaccines. Volume distribution (Figure 7B) indicated that, in VG and U3, unlike other vaccines, there were two particle populations distinguishable by volume.

## 4. Discussion

Vaccination still remains the best protection currently available against seasonal influenza [3]. Among the many types of seasonal influenza vaccines, the most widely used are inactivated influenza vaccines (IIVs) based on influenza virus propagation in chicken embryos [7]. Nevertheless, there are differences in the processes used by manufacturers to obtain the final product, especially at the multistep stage of downstream processing [12]. Annual updates of the WHO recommendations on influenza virus strains to be used in seasonal influenza vaccines and shortened production periods create additional difficulties in maintaining the consistency of vaccine formulations from year to year [11]. The standards for controlling the composition of inactivated influenza vaccines used today depend on the presence of reference reagents; they consider only a few key components, and do not allow for complete characterization of influenza vaccine composition [14]. Possible undetectable variations in the complex composition of inactivated influenza vaccines may affect the safety, immunogenicity, and ultimately the efficacy of these vaccines.

To address the above challenges, this paper first describes a comparative analysis of commercially available inactivated seasonal influenza vaccines (IIVs) included in the Russian National Immunization Program schedule. It studies the proteomes and molecular morphology of vaccines using a holistic approach including such methods as SDS-PAGE and sample deglycosylation, LC-MS/MS, and dynamic light scattering in combination with transmission electron microscopy. The methods used do not depend on reference reagents and therefore allowed for a quick and informative post-marketing study of IIVs.

For the general qualitative and quantitative characterization of the protein profiles of IIVs, we used standard methods: total protein quantitation by Lowry protein assay with precipitation and the SDS-PAGE assay. These methods for analysis of vaccines licensed in Russia were shown to have limited applicability. Synthetic polymers present in the SGU and SGF vaccines (SOVIDON^ТМ^: a copolymer of 2-methyl-5-vinylpyridine and N-vinylpyrrolidone) and GP (Polyoxidonium^®^: high-polymeric units of 100 kDa based on both N-oxide 1.4-ethylene piperazine and (N-carboxyethyl-) 1.4 ethylene piperazine bromide) interfered with total protein quantitation by Lowry protein assay (Figure 1) and protein separation by SDS-PAGE (Figure 2).

In vaccines without synthetic polymers (U3, U4, and VG), we revealed similar patterns of electrophoretic migration of proteins in gels. Major protein bands on the gels obtained corresponded in terms of molecular weight with influenza virion proteins: HA (cleaved into HA1 and HA2 subunits after reduction), NP, and M1 (Figure 2). Thus, we reproduced the results of previous studies based on SDS-PAGE analysis of inactivated influenza vaccines [12,35] and purified influenza virus virions [23,32] in relation to IIVs selected for study in this paper.

Since the influenza virus HA is an intensely N-glycosylated glycoprotein, the peptide N-glycosidase F (PNGase F) treatment of vaccine samples before application on gels leads to carbohydrate cleavage from this protein and consequently to increased electrophoretic mobility of HA1 and HA2, and to decreased fuzziness of the respective protein bands [15]. After deglycosylation, HA1 and HA2 bands may be reliably separated from NP and M1, respectively. This fact was used in a number of studies for absolute quantitation of HA content in vaccines using SDS-PAGE assay in combination with densitometry [11,15,26]. In particular, Li et al. [35] showed that this approach yielded a result that deviated from SRID by 12% to 22%.

In this paper, the PNGase F treatment of vaccine samples expectedly increased electrophoretic mobility of HA1 and HA2 (Figure 2). Moreover, after deglycosylation of HA1, the subunits formed several bands within the 37 to 45 kDa range. This may have been caused by the presence of several HA1 proteoforms corresponding to different types and subtypes of HA, as well as heterogeneity of HA glycosylation described in previous studies [43].

Using the results of gel densitometry after deglycosylation (Figure S1, and Tables S3 and S4) and our measured total protein concentrations (Figure 1), we calculated total HA concentrations in the vaccines without synthetic polymers. The approach used was that developed by Harvey et al. [23]. The obtained total HA concentrations in the vaccines without synthetic polymers ranged from 69.23% to 75.71% of the normative values specified in instructions for the preparations (Table 2). HA concentrations quantified by SDS-PAGE in our study could have been underestimated due to incomplete deglycosylation and presence of proteins with molecular weights beyond the studied molecular weight range. This indicated the need to optimize the deglycosylation conditions and to select conditions for SDS-PAGE to accurately quantify the HA content by SDS-PAGE. However, this was not among the aims of our study.

For deeper and more sensitive analysis of IIV protein composition, we used the LC-MS/MS assay. As has been shown in a number of studies, this assay provides an effective platform for identification and quantitative analysis of proteins in isolated influenza virus virions and influenza vaccines [16,17,32,44]. In addition, LC-MS/MS that was based on other physical processes than the Lowry assay and SDS-PAGE, theoretically allowed us to overcome the inapplicability of the Lowry assay and SDS-PAGE for the study of vaccines containing high-molecular-weight artificial polymers (SGU, SGF, GP).

The accuracy of label-free LC-MS/MS-based quantitation of protein concentrations in a complex mix is much lower than that of targeted approaches: mass spectrometry using synthetic isotope-labeled peptides (isotope dilution mass spectrometry (IDMS), high-resolution (HR) LC-MS) [18,19,22], and serological methods SRID [45] and ELISA (enzyme-linked immunosorbent assay) [46]. However, in comparison with the targeted methods, label-free LC-MS/MS did not depend on a potentially long and expensive stage of preparation of special reagents for quantitation of specific proteins (i.e., SIL peptide synthesis with a specific amino acid sequence and production of specific antibodies). Therefore, LC-MS/MS is much more appropriate for post-marketing control of IIVs given the annual update of their strain composition and, accordingly, annual update of control reagents. Although this approach is quite expensive today, we hope that it will become cheaper in the near future, as mass spectrometry equipment will become more available in many protein research laboratories.

In a paper recently published by Hawksworth et al. [17], internal protein standards were used to verify label-free protein quantitation (iBAQ) [27] of substances representing different stages of downstream processing in live-influenza vaccine production. Following this approach, we used internal standards to verify iBAQ-based protein quantitation in commercially available IIVs. The procedure used in our study was based on measurement of absolute protein concentrations using high-resolution (HR) LC-MS [22]. Verification of the iBAQ-based evaluation allowed us to perform reliable quantitative proteomic analysis of IIVs. In particular, we could evaluate the relative content of cell substrate proteins in the vaccines (chicken (*Gallus gallus*)), and HA A and B, NA, M1, NP, and other structural proteins of influenza viruses. In additionally demonstrating the correctness of the iBAQ-based assessment of the HA content in IIVs, we showed that, in vaccines without synthetic polymers (U3, U4, VG), the total HA content quantified using the LC-MS/MS assay correlated with SDS-PAGE-based measurements (Table 4).

Host cell proteins are an unavoidable component of any viral vaccine. Complete removal of substrate proteins is impossible not only due to technical limitations of the methods for purification of the virus-containing liquid, but also because of incorporation of host proteins into the structure of the influenza virus virion [32]. However, downstream processing of IIVs (Table S1) is aimed at eliminating substrate proteins (chicken proteins) from a final product. Removal of chicken proteins is required to prevent exceeding the maximal level of total protein (no more than 300 μg per dose) [14] and to minimize the concentration of the potential allergen, ovalbumin (no more than 1 μg per dose) [46,47] in the final product. We found that, in the U3, U4, SGU, SGF, and VG vaccines, the relative proportion of chicken proteins (by substance) varied from 18% (SGU) to 36% (SGF). The GP vaccine contained 80% of chicken protein, falling out of said interval, which may raise concerns about the safety of this vaccine compared to the rest of the vaccines studied.

Downstream processing of the virus-containing allantoic fluid (VCAF; Table S1) in the IIV production process is usually designed to enrich the preparation with the main surface glycoprotein of the influenza virus HA [12]. This is required to guarantee the vaccine’s ability to stimulate a sufficiently high level of neutralizing antibodies to HA and to form sterilizing immunity as a result. From this point of view, abundance of non-HA (or other) viral proteins, in comparison with the abundance of HA, reflects the success of a strategy for vaccine enrichment with hemagglutinin. However, much of the currently available experimental evidence was on the role of non-HA proteins in protective immunity against influenza [15]. Considering this, one of the aims of our study was to quantify non-HA viral proteins in different IIVs. This issue is also important in light of the fact that existing WHO recommendations on the composition and quality control of influenza vaccines require manufacturers to control only the HA content (among all viral proteins), setting SRID as a control method [14]. This method, in addition to its dependence on reference reagents, is difficult to adapt for studies involving low-represented proteins in IIVs, such as NA and other viral proteins. Therefore, the LC-MS/MS assay represents an alternative to SRID, allowing for the use of reference reagents to detect low-presenting proteins in IIVs and, thereby, obtain more detailed information about vaccine composition.

We could reliably detect NA, M1, and NP proteins in all the studied IIVs. Relative abundance of NA varied slightly between the vaccines. A significant scatter was found in the M1 and NP contents of different preparations. This finding is consistent with results of non-HA protein quantitation in commercially available vaccines using Western blot analysis. Differences in the non-HA protein concentration correlate with the intensities of cellular and humoral immune responses [15]. Thus, our study indicated the potential variability of commercially available IIVs licensed in Russia by their ability to elicit an immune response to non-HA proteins of the influenza virus, and, therefore, potentially stimulate development of cross-protective immunity against various types and subtypes of the influenza virus.

It is worth mentioning that not only proteins but also other biological molecules, e.g., nucleic acids or lipids may affect the vaccine efficiency, boost unspecific immune response, or induce undesirable side-effects [8,48]. That is why a comprehensive investigation of modern vaccine preparations using new sophisticated methodologies is of crucial importance. At the moment, the quality assurance/quality control protocols applied for IIV in Russia do not include testing for RNA (e.g., by applying reverse transcription polymerase chain reaction, RT-PCR). Yet, future progress in vaccinology science as well as the demands of vaccinology practice will definitely allow one to provide a comprehensive control of different constituents within the vaccine preparations to understand the responsible factors better.

Dynamic light scattering was used to measure the vaccine-particle diameter and to assess vaccine-suspension uniformity. According to Kon et al. [12], particle size distribution in split virion IIVs was most affected by the virion cleavage stage (increasing vaccine preparation dispersion) and the subsequent sterile filtration stage (decreasing preparation dispersion and removal of the coarse-particle fraction (≥200 nm)). Therefore, the significant differences in particle distribution found in our vaccines suggest there are differences in the raw virus-containing fluid-processing technologies used by vaccine manufacturers (Figure 7).

Electron microscopy showed that particles detected by dynamic light scattering were mainly molecular aggregates containing HA (rosettes). As shown by McCraw et al. [40], HA aggregation into rosettes occurs through hydrophobic interaction among HA transmembrane domains. The presence of HA rosettes in split virion IIVs, similar to those in purified recombinant HA preparations, is understandable in terms of a typical production strategy for this type of vaccine [12]. During the vaccine production process, the influenza virus is cleaved by detergents (for example, octyl glycoside or Triton X-100), destroying the lipid membrane of the virus and transferring the integrated proteins (HA, NA, M2, NB) into the solution. During downstream processing, the concentration of detergents is critically reduced as a result of fractionation and dilution of the liquid containing inactivated cleaved virions. Removal of detergents from the solution can theoretically lead to protein aggregation through hydrophobic interprotein interactions. Experimental results obtained by Koroleva et al. [15] also agree with this assumption. Using coimmunoprecipitation, the authors detected aggregation of heterologous HA proteins, as well as M1 and NA proteins with HA protein in licensed split virion IIVs. Thus, the abundance of molecular aggregates we observed in the studied vaccines is consistent with published results (Figure 6).

## 5. Conclusions

This work allowed us to collect a large amount of data on proteomes and molecular morphology of inactivated split vaccines for the prevention of seasonal influenza included in The National Immunization Program Schedule in Russia. The comparison of different preparations revealed that vaccines Ultrix^®^ and Ultrix^®^ Quadri produced by FORT and the trivalent vaccine Vaxigrip^®^ produced by Sanofi Pasteur contain fewer admixture quantities of host cell proteins, demonstrate good correlation among mostly abundant viral proteins detected by different methods, and have well-organized antigen rosettes that might be favorable for triggering better immune response. Based on the evidence found, we can conclude that the complex analytical approach tested in our work, which includes label-free quantitative LC-MS/MS of protein mixtures based on the iBAQ data processing algorithm, in combination with deglycosylation of the proteins before the analysis, SDS-PAGE, DLS and EM, appears to be informative, productive and suitable for independent post-marketing control of commercially available inactivated split vaccines for the prevention of seasonal influenza in their final formulation.

## Figures and Tables

**Table 1:** Theoretical ratios of control proteins and corresponding stable-isotope-labeled (SIL) peptides injected into each vaccine sample *.

Vaccine	APOA	APOB	FIBB	FIBG
U3	12:12	0.3:2.5	3:8	4:4
U4	12:12	0.3:2.5	3:8	4:4
SGU	15:12	0.3:2.5	5:8	4:4
SGF	25:12	0.5:2.5	5:8	8:4
GP	2.5:12	0.01:2.5	0.5:8	0.5:4
VG	15:12	0.1:2.5	4:8	4:4

Notes: * theoretical ratio of control protein to corresponding SIL peptide added per 0.5 μg of total vaccine protein, pmol control protein:pmol SIL peptide; APOA, apolipoprotein A1; APOB, apolipoprotein B; FIBB, fibrinogen beta chain; and FIBG, fibrinogen gamma chain.

**Table 2:** Recovery of total HA concentration by PNGase F treatment and SDS-PAGE compared with the normative HA concentration.

Vaccine Sample	U3	U4	VG
Total protein concentration, μg/mL	99.89	178.11	159.86
Relative HA weight, %, based on SDS PAGE	62.37	49.92	42.62
HA concentration based on SDS PAGE, μg/mL	62.31	88.91	68.13
Normative HA concentration (based on SRID), μg/mL	90.00	120.00	90.00
Recovery, %	69.23	74.10	75.71

Notes: For U3, U4, and VG, the image in Figure 2B was used for analysis; total protein concentration—our protein measurements (Figure 1); HA content based on SDS-PAGE measured as the HA proportion using the weight of total protein concentration; and recovery (%)—ratio of HA concentration measured by SDS-PAGE to normative HA concentration expressed as a percentage.

**Table 3:** HA and neuraminidase (NA) strain identification and sequence coverage in IIVs based on LC-MS/MS assay.

Protein	Coverage, %					
	U3	U4	SGU	SGF	GP	VG
HA A/Brisbane/02/2018	31.4	34.3	12.0	32.0	5.5	28.3
HA A/Kansas/14/2017	46.5	50.0	16.0	33.7	12.2	37.5
HA B/Colorado/06/2017	37.2	44.9	19.0	42.2	11.7	29.2
HA B/Phuket/3073/2013	-	47.8	-	-	-	-
NA A/Brisbane/02/2018	22.8	20.7	8.0	18.8	6.2	18.1
NA A/Kansas/14/2017	40.7	37.1	11.0	27.7	10.2	33.3
NA B/Colorado/06/2017	35.4	21.9	9.0	27.5	5.6	26.4
NA B/Phuket/3073/2013	-	25.5	-	-	-	-

Note: Coverage (%) is the percentage of peptide coverage of theoretically predicted amino acid sequences of proteins identified via MS/MS.

**Table 4:** Comparison of absolute quantitation of total HA by SDS-PAGE and by label-free LC-MS/MS.

	U3	U4	VG
HA concentration by SDS PAGE, μg/mL	62.30	88.91	68.13
HA concentration by LC-MS/MS, μg/mL	58.12	82.23	64.84
Relative difference, %	6.72	7.51	4.83

Note: Relative difference (%) is the ratio of the difference between total HA concentrations quantified with SDS-PAGE and with LC-MS/MS to total HA concentration quantified with SDS-PAGE.

**Table 5:** Recovery of total HA concentration by LC-MS/MS compared to the normative HA concentration.

	U3	U4	VG
HA concentration based on LC-MS/MS, μg/mL	58.12	82.23	64.84
Normative HA content, μg/mL	90.00	120.00	90.00
Recovery, %	64.57	68.53	72.04

Note: Recovery (%), ratio of total HA concentration measured by LC-MS/MS to normative concentration of total HA.

**Table 6:** DLS results and polydispersity index calculation.

Sample	U3	U4	SGU	GP	VG
**PDI**	0.283	0.328	0.191	0.391	0.54
**PDI SD**	0.002	0.025	0.004	0.006	0.009
**SNR**	0.897	0.917	0.901	0.876	0.893
**SNR SD**	0.00199	0.00117	0.00244	0.00735	0.00264

Note: PDI, polydispersity index; SD, standard deviation; SNR, signal-to-noise ratio.

## Supplementary Materials

The following are available online at https://www.mdpi.com/2076-393X/8/3/488/s1. Supplementary Document 1: figures and tables, Figure S1: SDS-PAGE images of vaccine preparations under reducing conditions (A) without and (B) with the preliminary deglycosylation of proteins; Figures S2–S19: chromatograms of extracted isotopically labeled peptides and natural ions for control proteins injected in vaccine samples; Figure S20: Verification of label-free LC-MS/MS-based quantitation of proteins in vaccine samples; Figure S21: EM images of influenza virus virion from (A) the pooled harvest fluid, (B) hemagglutinin aggregates in the U4 sample, and (C) magnification of one hemagglutinin aggregate from B; Table S1: general steps in the downstream processing of split-virion inactivated influenza vaccines (IIVs); Table S2: Composition of LC-MS quality control mix. Table S3: densitometry results of protein bands in the gel image for vaccine preparation without the deglycosylation step. Band numbering corresponds to that in the gel image in Figure S1A; Table S4: densitometry results of protein bands in the gel image at 15% concentration of polyacrylamide in a reducing gel. Band numbering corresponds to that in the gel image in Figure S1B); Supplementary Document 2: MaxQuant report_U3; Supplementary Document 3: MaxQuant report_U4; Supplementary Document 4: MaxQuant report_SGU; Supplementary Document 5: MaxQuant report_SGF; Supplementary Document 6: MaxQuant report_GP; and Supplementary Document 7: MaxQuant report_VG.
